# Interactive Apps Promote Learning of Basic Mathematics in Children With Special Educational Needs and Disabilities

**DOI:** 10.3389/fpsyg.2018.00262

**Published:** 2018-03-06

**Authors:** Nicola J. Pitchford, Elizabeth Kamchedzera, Paula J. Hubber, Antonie L. Chigeda

**Affiliations:** ^1^School of Psychology, University of Nottingham, Nottingham, United Kingdom; ^2^School of Education, Chancellor's College, University of Malawi, Zomba, Malawi

**Keywords:** special educational needs and disabilities, interactive apps, tablet technology, primary school, mathematics

## Abstract

Interactive apps delivered on touch-screen tablets can be effective at supporting the acquisition of basic skills in mainstream primary school children. This technology may also be beneficial for children with Special Educational Needs and Disabilities (SEND) as it can promote high levels of engagement with the learning task and an inclusive learning environment. However, few studies have measured extent of learning for SEND pupils when using interactive apps, so it has yet to be determined if this technology is effective at raising attainment for these pupils. We report the first observational study of a group of 33 pupils with SEND from two primary schools in Malawi that are implementing a new digital technology intervention which uses touch-screen tablets to deliver interactive apps designed to teach basic mathematical skills. The apps contain topics that align to the national curriculum. To assess learning gains, rate of progress (minutes per topic) for each pupil was determined by calculating the average time taken to complete a topic. Progress rate was then correlated with teacher ratings of extent of disability and independent ratings of pupil engagement with the apps. Results showed SEND pupils could interact with the apps and all pupils passed at least one topic. Average progress rate for SEND pupils was twice as long as mainstream peers. Stepwise regression revealed extent of disability significantly predicted progress rate. Further exploratory correlations revealed pupils with moderate to severe difficulties with hearing and/or language made slower progress through the apps than those with greater functionality in these two domains because the use of verbal instructions within the apps limited their capacity to learn. This original quantitative analysis demonstrates that interactive apps can raise learning standards in pupils with SEND but may have limited utility for pupils with severe difficulties. Software modifications are needed to address specific areas of difficulty preventing pupils from progressing.

## Introduction

We investigated if a novel and innovative technology-based education intervention is suitable for supporting the development of early mathematical skills in pupils with Special Educational Needs and Disabilities (SEND). The intervention is currently being trialed across Malawi, a low-income country in Sub-Sahara Africa, and has been shown to be highly effective at supporting the acquisition of basic mathematical skills with mainstream children in Malawi and the UK (Pitchford, 2015; Outhwaite et al., 2017; Pitchford and Outhwaite, 2017). As the project scales within Malawi to reach all primary schools (Hubber et al., 2016), in line with the United Nation's Sustainable Development Goal 4 to “ensure inclusive learning and equitable quality education and promote lifelong learning for all” (United Nations, 2016, p. 5), it is now timely to consider if this innovative technology can also enhance the education of children with SEND to help them reach their full potential.

Pitchford (2015) conducted a pupil-level randomized control trial in a primary school in Malawi with mainstream children attending the first 3 years (standards) of compulsory education, to establish proof of concept that this digital education technology intervention could be effective at raising learning outcomes in Malawi—a country with a history of poor educational attainment and high innumeracy and illiteracy rates (Kadzamira and Rose, 2003). By the end of primary school, <50% of Malawi children have achieved basic competency in mathematics and reading (Milner et al., 2011). The technology intervention utilizes hand-held tablets to deliver a series of child-centered, self-paced, interactive apps designed to support the acquisition of basic mathematical skills in mainstream pupils. Pupils received the technology intervention on a daily basis in a purpose-built Learning Center—a small classroom separate from the rest of the school. Class teachers implemented the technology intervention to groups of 30 children at a time. Technical support was provided by the Voluntary Service Overseas (VSO) to teachers implementing the intervention but teachers were instructed to offer no pedagogical support to children whilst interacting with the apps. Children were assessed on psychometric measures of mathematical ability before and after the intervention period. This enabled performance gains to be quantified over the intervention period and compared across groups of children receiving the technology intervention (intervention group) or regular teacher-led mathematics tuition (control group). Performance gains were taken as a direct measure of learning. Results showed after just 8 weeks of intervention with the technology, pupils in the first 3 years of primary school in Malawi significantly outperformed those following standard teacher-led mathematics instruction. This could not be attributed to novelty effects of using the tablet technology as the study included a placebo group of pupils from the same school who accessed the touch-screen tablets with the same dosage as the intervention group but the placebo group interacted with design apps rather than maths apps. Results showed the placebo group made similar gains in mathematics over the duration of the intervention as pupils in the control group, who received standard teacher-led practice. This demonstrates that the tablet technology alone did not contribute toward the higher maths performance found at post-test in the intervention group, but rather the maths apps that the pupils interacted with throughout the intervention were responsible to improving learning outcomes.

As the intervention scales across Malawi for mainstream pupils it is also being delivered pupils with SEND. It is therefore necessary to evaluate the effectiveness of this intervention for pupils with SEND as this intervention was not designed specifically for SEND pupils, many of who have specific motor and sensory processing difficulties that might make interacting with this technology problematic. The National Special Needs Education Policy of Malawi describes learners with special educational needs as those “who require special service provision and support in order to access education and maximize the learning process” (Ministry of Education, 2007, p. 6). According to the Ministry of Education in Malawi (2007), pupils with SEND include children who fall into any of the following categories: sensory impairment (vision, hearing, deaf-blind); cognitive difficulties (intellectual, specific disabilities, and gifted and talented); socio-emotional and behavioral difficulties (autism, hyperactivity, and other vulnerable children); and physical and health impairments (spina bifida, hydrocephalus, asthma, and epilepsy). Approximately 2.3% of children at primary school in Malawi are registered with SEND (Munthali, 2011). The intervention being evaluated in this study requires a high level of sensory processing (visual, auditory, and kinaesthetic) and manual coordination and dexterity to interact with the apps, so some SEND pupils with difficulties in these domains might find interacting with the apps problematic, without modification of the software. As the intervention has been designed to support development of numeracy skills in mainstream pupils, it is therefore vital to assess its suitability for SEND pupils, as software modification may be required for maximize learning potential for SEND pupils, especially those with sensory processing and/or motor difficulties.

High-quality education is deemed critical in helping individuals with SEND to maximize their full potential. However, as the Convention on the Rights of Persons with Disabilities (United Nations, 2007) articulates, there is a complex interaction between individuals with SEND and attitudinal as well as environmental barriers that hinders their full participation in society on an equal basis with others. In some circumstances, education can be a barrier for children with SEND participating in society if the country's educational system is not sufficiently geared toward supporting these individuals. Attitudes of teachers can impact both positively and negatively on how pupils with SEND progress through school, and attitudes toward disability in society can influence who gets access to education. Demonstrating that pupils with SEND can learn could be influential in changing attitudes which, in turn, could encourage more SEND pupils to attend and stay in school. The technology intervention evaluated in this study enables learning to be monitored directly within the apps as children are required to pass a quiz at the end of each topic which requires application of knowledge taught to solve a series of novel questions. Progression throughout the app is not possible unless the quizzes are passed, so passing the quiz for a particular topic is a direct measure of learning. Quantifying progress, especially in relation to mainstream pupils accessing the same intervention, could be instrumental in changing attitudes toward SEND pupils' capacity to learn. This is particularly poignant in low-income countries, such as Malawi, where there are additional hurdles to accessing education, which result in many children with SEND being kept at home.

Within Malawi, barriers to quality education for children with SEND include poverty, large pupil-to-teacher ratios, distance from home to school—which for families of children with mobility difficulties can be particularly problematic as many do not have wheelchairs or other mobility aides, inadequate learning materials (Hughes et al., 2016), and discrimination within the community (Kelly et al., 2012). If parents of children with SEND are ashamed of their child's disability, or do not believe their child is capable of learning, and if there are few opportunities for employment of individuals with SEND upon completion of schooling, parents may opt to withdraw their child from the education system (Mutua and Dimitrov, 2001). It is therefore critical to demonstrate that children with SEND can learn, so they are given the opportunity to access primary education that is provided by the state and teaches fundamental skills, such as numeracy and literacy, which form the bedrock of later learning. Mobile technologies that deliver apps designed to support the acquisition of basic numeracy and literacy skills in a personalized, self-paced, manner, with minimal need for specialized adult support, could be particularly useful for children with SEND in accessing quality instruction, as they can be deployed in the home as well as the school environment (Melhuish and Falloon, 2010). Such technologies could be particularly advantageous for children with severe mobility difficulties that prevent them from attending school on a regular basis.

Inclusive education interventions are needed to address these barriers and help change attitudes toward the learning capabilities of children with SEND. Within Malawi, to embrace inclusive education, children with SEND are enrolled in mainstream schools, as advocated by international standards and frameworks such as the Salamanca Statements and Framework for Action on Special Needs and Education (UNESCO, 1994) and the Convention on the Rights of Persons with Disabilities (United Nations, 2007). Although the Malawi government requires all teacher training colleges to include SEND training in their courses for pre-service trainee teachers, few institutions offer specialized SEND training programmes (Chitiyo et al., 2015). There are therefore currently insufficient specialist teachers to meet the growing number of pupils identified with SEND entering the school system (Hughes et al., 2016) and there is no specific curriculum for SEND pupils (Chitiyo et al., 2015). As a result, pupils with SEND face many challenges to learning in mainstream schools when there are no specialist teachers or materials to support their needs. The intervention evaluated in this study requires little specialist adult support to implement, as children work on a one-to-one basis with the technology and a virtual teacher within the apps demonstrates “how to” perform certain tasks before children practice a particular topic. Instructions can be repeated upon demand, as often as needed, so this affords personalized, self-paced, learning, without the need for specialist adult support.

Tablet technology could help to address some of the shortfalls within the current education system within Malawi for pupils with SEND. Touch-screen tablets, such as iPads, are light-weight and portable, have a long battery life and screen size appropriate for young children, and do not rely on additional dexterity devices, such as keyboard and mouse (Kucirkova, 2014), making them particularly suited to pupils with SEND. In addition, they can store multiple child-friendly educational apps hosting software features that place the child in active control of their learning. Specifically, apps that include multiple representations of information, such as pictures, video, and animation, varying or progressive levels of task difficulty, clear goals and rules, learner control, task feedback, and repetition, can serve to create an individualized learning environment (see also Rose et al., 2005; Condie and Munro, 2007), enabling children to progress at their own pace. Whilst compelling, few studies to date have investigated systematically if this technology can assist pupils with SEND in acquiring basic skills, such as numeracy or literacy.

Tablet technology can also offer a vehicle for engendering an inclusive learning environment when used with SEND pupils alongside their mainstream peers. Kagohara et al. (2013) reported that SEND pupils willingly adopt this technology, such as iPads, which is perceived as socially more acceptable and less stigmatizing than previous forms of assistance technology, such as adjustable keyboards, which are bulky and awkward to use (Flewitt et al., 2014). Moreover, due to their portability and long battery life, touch-screen tablets can offer a bridge between the school and home environment, which could be highly beneficial for a low-income country like Malawi, where many pupils with SEND experience difficulties in getting to school and grid supply of electricity is often lacking or unreliable in villages and homes. The current intervention utilizes solar panels to charge the devices overnight, providing a sustainable and reliable electricity source, which could impact on communities in Malawi by providing dependable home-school links. Strnadova and Cumming (2013) studied the use of iPad-based interventions across home-school settings and reported pupils with SEND experienced positive teaching and learning, higher engagement with educational tasks, and closer home-school links. This raises the possibility that tablet technology could act to breakdown some of the sociocultural barriers concerning attitudes toward the educational capability of pupils with SEND, especially in low-income countries such as Malawi.

Despite the potential for tablet technology coupled with high-quality educational apps to support the learning of pupils with SEND, there is little empirical evidence demonstrating that this technology is an effective means of instructional delivery for raising learning standards (Chmiliar, 2017). A recent systematic review of the existing evidence of the effectiveness of touch-screen tablets in primary and secondary schools for raising learning outcomes of mainstream pupils is limited and fragmented (Haβler et al., 2016). Studies that have measured learning outcomes of pupils with SEND, by tracing progress quantitatively whilst working through educational apps, are scarce. Most studies exploring the utility of touch-screen tablets with SEND pupils are qualitative in nature (Khoo, 2017) or report on testimonials from parents and educators as to their effectiveness (Shah, 2011). These exploratory studies have indicated that tablet technology can provide an effective means of learning support for pupils with SEND, especially in the early years (see Chmiliar, 2017), but this needs to be validated by studies that quantify learning gains.

Thus, it remains to be determined if educational apps can be utilized effectively by pupils with SEND to raise attainment in basic skills. This type of instruction may pose particular challenges to pupils with physical and sensory difficulties, such as difficulties with manual coordination and/or hearing and visual impairments, as multisensory, interactive, apps require visual, auditory, and kinaesthetic processing (including hand-eye coordination and manual dexterity) to interact successfully with the software. In addition, some apps, such as those evaluated in this study, require pupils to attend to visual demonstrations and understand verbal instructions so pupils with attentional, language, and/or intellectual difficulties may struggle to engage with the software. Although most previous research reports SEND pupils have positive experiences of using touch-screen tablets and high levels of engagement (Strnadova and Cumming, 2013; Chmiliar, 2017; Khoo, 2017) if they find interacting with the technology difficult, because of their disabilities, they may not enjoy this type of instruction so could disengage with the learning process. Assistive aids, either built into the tablet technology or external to the technology, can be used to overcome some of these difficulties (Dell et al., 2017), but these are not always available in low income countries, such as Malawi.

## Current study

We explored if a novel educational technology intervention that is being implemented in over 100 mainstream primary schools across Malawi by the VSO, in their flagship “Unlocking Talent Project” (see https://unlockingtalent.org/), is suitable for pupils with SEND. We report on data from a group of standard 1 children (*N* = 116) taking part in this project in the sections below.

## Unlocking talent intervention

**Figure 1:**
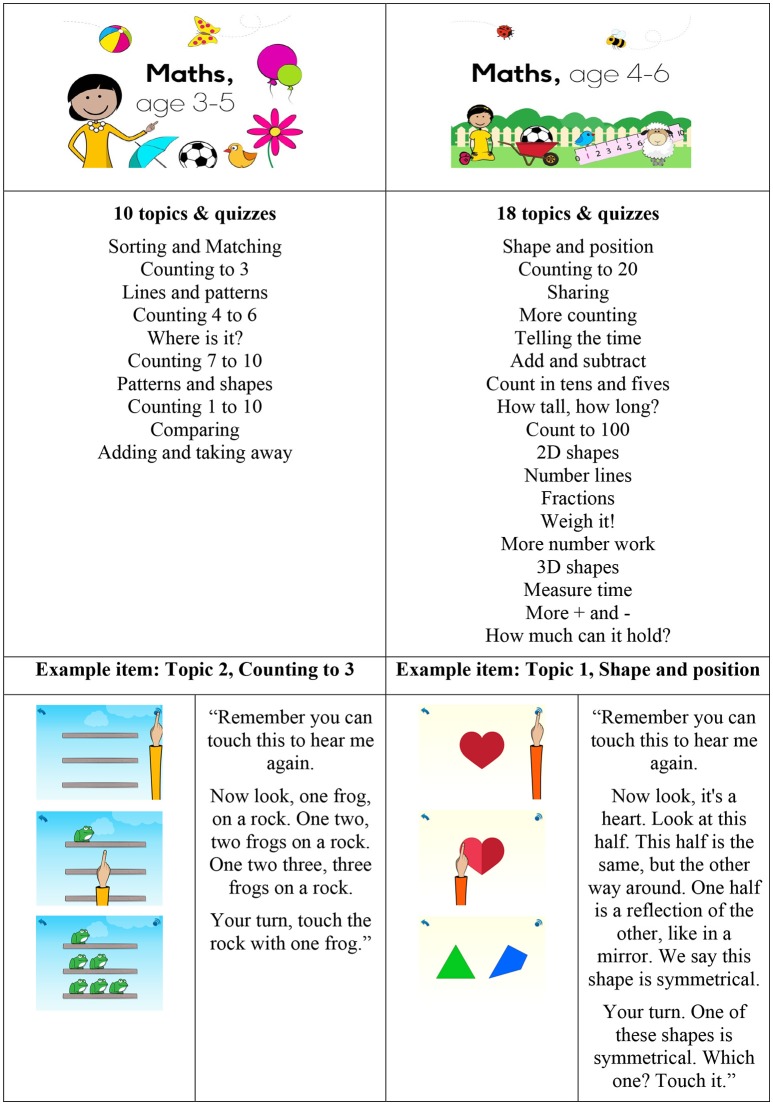
Topics covered in the onebillion maths apps and example items.

The intervention uses touch-screen tablets to deliver a series of child-centered, interactive, apps that teach early numeracy skills. The apps employed in this project have been designed and developed by the charity *onebillion*—finalists in the Global Learning Xprize (see https://onebillion.org/). They cover key topics that align with the national primary school mathematics curriculum, presented in staged sequence, that enable children to build on previous knowledge (Magliaro et al., 2005), and extend their knowledge beyond their current ability level (Inal and Cagiltay, 2007). Topics covered in the apps are listed in Figure 1.

Children interact with the apps on a one-to-one basis through headsets connected to a tablet and instructions are delivered in their own language, Chichewa. Efficient and effective delivery of one-to-one instruction has been to shown to be an important component of math interventions (Holmes and Dowker, 2013). Content is provided simultaneously in both visual and auditory modalities. Multisensory input, such as this, is known to support children's understanding when acquiring new skills (Pavio, 1986; Carr, 2012). Children are required to interact with the app content directly through manipulation of virtual objects, verbal labels, and numerical representation which fosters active learning (Lindahl and Folkesson, 2012). A virtual teacher demonstrates how to perform different tasks within a topic then children practice each of the different tasks included in a topic. Whilst practicing the tasks formative feedback (positive and negative) is given after every interaction with the software and children can repeat instructions as often as they require, enabling children to progress at their own pace. This provides effective scaffolding for pupils with differing needs and creates an individualized learning environment (Slavin and Lake, 2008; Gulliford and Miller, 2015) that promotes learner autonomy, which has been shown to be effective for improving educational outcomes (Morrison et al., 1992).

Learning within the apps is measured by topics passed (number of certificates awarded). At the end of each topic children are presented with a quiz built into the software that assesses their understanding of the mathematical concepts taught in that topic. Each quiz presents questions similar to those practiced within a particular topic but uses new stimuli and questions that require application of knowledge acquired to novel items to succeed. As such, the quizzes engender retrieval-based learning which is known to improve learning outcomes (Karpicke and Grimaldi, 2012; Dunlosky et al., 2013). No feedback is given within the quiz so children do not know if they have answered a quiz question correctly or incorrectly until the end of the quiz, when the number of correct answers is released. Children are required to pass the quiz with 100% accuracy before proceeding to the next topic. Accordingly, passing a quiz is a direct measure of learning and children cannot progress within the apps until they have passed the quiz for a particular topic. Children are awarded with a certificate for each quiz they pass, and the number of certificates awarded to each child is recorded within the software, providing an opportunity to use this data for monitoring individual pupil's progress.

Within the SAMR Model of integrating technology into the classroom (Puentedura, 2012), these apps operate at the Modification level. They allow for “significant task redesign” through the interactive features and individualized feedback that enables children to discover possible outcomes through exploration of manipulatives (virtual objects), verbal labels, and numerical representations, presented within the apps, and enable progression through the apps at the child's own pace. Thus, within the SAMR Model, these apps can be considered transformational, although they do not operate at the highest level of Redefinition in that they do not allow for “creation of a new task, that was previously inconceivable.” Mobile learning is proposed to be characterized by three key features. It is considered to be (1) personal and personalized, (2) situated across contexts and time, and (3) connected to information, people, and practices (Romrell et al., 2014). Whilst these apps are “personalized” as children can progress through the apps at their own pace within the school environment, they are not “situated” as in Malawi children access the apps is within the Learning Center only, and they are also only “connected” through provision of aggregated monitoring data to educators within Malawi but children are not allowed access to the internet to other sources of information. Rather, the apps' features draw on characteristics of instructional psychology by combining properties of direct instruction (Kirschner et al., 2006), for example, feedback, repetition, and reward, with features of free play (Gray, 2015) particularly self-regulation, to deliver a child-centered-by-scaffolded learning environment (Mayer, 2004).

Whilst the intervention is being scaled across Malawi and the scale-up is being evaluated (Hubber et al., 2016), class teachers implement the intervention with the assistance of a VSO volunteer. The teachers receive training from VSO in how to use the technology, including how to turn on and turn off the tablets, control the volume, connect the headphones, charge the tablets, and register pupils on the apps. During the sessions, they assist pupils with using the technology, such as adjusting the volume of the headphones if a pupil cannot hear the instructions given within the apps, or restarting the apps if they freeze. They are instructed to offer little pedagogical support and not to complete trials within the apps as these are used to monitor pupil progress. If a child is struggling with a particular topic the teacher or assistant might take off the child's headphones, listen to the instructions themselves, and explain the task to the child, before replacing the child's headphones and encouraging them to continue working through the topic. Class teachers of mainstream pupils find this intervention easy to implement and enjoy observing their pupils learning with this technology (Pitchford, 2015). When implemented with SEND pupils, the specialist SEND teacher, if available in school, implements the intervention with the assistant of a parent or VSO volunteer.

Whilst these interactive, child-centered, apps have been shown to be highly effective in promoting learning of mainstream children (Pitchford, 2015; Outhwaite et al., 2017), it is yet unknown if they can be utilized effectively by pupils with SEND. Engagement with the apps requires multisensory processing, as information is presented in both the aural and visual domain, and children are required to make motoric touch-screen responses, including motor precision to isolate objects and motor coordination to drag and drop objects. The speed and accuracy with which pupils interact with the apps determines their rate of progress. Accordingly, a high level of attention is needed to progress successfully through the apps. Whilst this technology is engaging for mainstream pupils, and has been shown to increase attentional skills in addition to mathematics knowledge (Pitchford and Outhwaite, under review), the requirement for a high level of multisensory processing may render this intervention unsuitable for pupils with SEND.

We conducted a quantitative, observational study, with a group of 33 SEND pupils attending either of two mainstream primary schools in Malawi. To assess learning gains, we obtained ratings from specialist teachers in these schools on extent of disability for each pupil in their care and we determined rate of progress through the apps with monitoring data recorded by the touch-screen tablets after each session. In addition, we recorded each pupil interacting with the apps in a short 2-min video and asked four professional educators to rate the videos on six behavioral measures needed to successfully interact with the apps, as outlined above. We used these data to ask: (1) How do pupils with SEND interact with the tablet technology and maths apps? (2) Can pupils with SEND make progress in learning maths with the apps? (3) How does extent of disability and interactions of SEND pupils with the technology relate to their progress in learning maths?

## Methods

### Design

This observational study employed a correlational design to investigate associations between extent of disability exhibited by pupils with SEND, their ability to interact with the tablet technology and maths apps being used in their school, and the learning gains they achieved through interacting with the technology. The National Commission for Science and Technology in Malawi granted ethical approval for this study. Opt-out parental consent was used to accommodate for the high rate of illiteracy in Malawi amongst the adult population. Opt-in parental consent is not suitable in Malawi and would lead to a highly biased sample. According to standard guidelines by the National Commission for Science and Technology, meetings were held with the parent association at each school who were informed of the study and acted on behalf of the parents of participating pupils. Parents were informed of the study through the parent association, and given the option not to include their child in the study. No parent chose to withdraw their child from the study. All data was password protected and accessible only to the research team.

### Participants

A total sample of 33 pupils registered at a SEND unit attached to one of two mainstream primary schools from the Unlocking Talent Project in Malawi participated in the study. All of the children registered with SEND at Lilongwe Demonstration School (LD, *N* = 16), an urban school in the capital and central district of Malawi with a total number of 2393 pupils ranging from standard 1 to standard 8, and St. Joseph's Demonstration School (SJD, *N* = 17), a rural school in the southern district of Malawi with a total number of 787 pupils ranging from standard 1 to standard 8, took part. The first author interviewed the specialist teachers at each of the two participating schools to gather details of the key areas of difficulty for each SEND pupil (see Appendix 1) and other characteristics, including age, gender, and years in school (see Table 1). Note: The Malawi education system is ability rather than age-based. Pupils are grouped into standards based on their overall ability not their age and pupils can repeat standards if they fail to progress. Thus, it is common for there to be a wide age range within a standard, as is reflected in our sample of SEND pupils (see Table 1 below).

**Table 1:** Summary of SEND pupil sample.

**Sample**	***N***	**% of SEND pupils in school**	**Gender (M:F)**	**Age in years mean (SD)**	**Years in school mean (SD)**	**Total disability score mean (SD)**
LD	16	0.67	9:7	10.56 (3.16)	2.73 (2.18)	4.81 (1.76)
SJD	17	2.16	12:5	9.18 (2.40)	1.94 (1.30)	4.00 (2.89)
Total	33	1.04	21:12	9.85 (2.84)	2.33 (1.80)	4.39 (2.41)

### Intervention implementation

Each of the SEND pupils that took part in this study accessed the series of maths apps developed by *onebillion* through daily sessions, as part of their usual mathematics instruction. Sessions took place within a dedicated “Learning Center” located within the school. The Learning Center hosted the touch-screen tablets (mini-iPads) used to deliver the apps, which were charged daily by solar panels attached to the roof of the Learning Center, and were kept in a secure cabinet overnight. The decision to use iPads in this intervention was made by *onebillion* because of the long battery life and high durability. Each session lasted for 1 h. Children used the tablets with a protecting casing whilst sat on the floor of the Learning Center covered in bamboo matting for extra protection against breakages. A SEND teacher and at least one volunteer managed the sessions and assisted pupils with using the technology (e.g., by adjusting the volume of the headsets) and progressing through the apps (e.g., by reminding children to press the “next” button to move onto the following task). Importantly, pupils were required to complete the trials within each task themselves, as monitoring data recorded within the apps was used to determine rate of progress. In this study, SEND pupils accessed the apps without the use of additional assistive technology aids.

### Materials and procedure

During January 2017, the first and third author visited LD and SJD on two occasions, to interview the specialist teachers and to obtain video-recordings of each SEND pupil interacting with the technology. The first author interviewed the specialist teachers to gather information of the main difficulties exhibited by each of the pupils with SEND in their care and to obtain ratings on the extent of disability for each pupil (see below). At the same time, the third author made a 2-min video recording of each SEND pupil interacting with the tablet technology and maths apps. The week prior to collecting the video data, SEND teachers at each school were informed that the videoing would take place on a particular day and were advised to deliver the session in the Learning Center as per usual. The third author videoed each SEND pupil attending that session in one 2-min video clip, moving systematically from pupil to pupil around the class. Only one 2-min video clip was recorded for each SEND pupil present in the learning center that day. The video clips focussed on the SEND pupils interacting with the touchscreen. This procedure ensured the videos were as representative as possible of SEND pupils interacting with the apps. The first author was not present when the video recordings were taken, so did not observe how individual pupils interacted with the apps during their sessions in the Learning Center. A Panasonic HC-V250 digital video-recorder secured to a Hama Star 700 tripod was used to make the video recordings. An anonymous participant identity number was assigned to each recording and recordings were stored as MPEG-4 images on a separate hard drive that was password protected. These video clips were used to generate ratings from four educators on the extent of interaction made with the technology, as described below. Three sets of data were obtained for this study.

### Specialist teacher ratings of pupil disability

Specialist teachers in each school were asked to rate the extent of disability for each pupil with SEND in their care. For each pupil registered in the SEND unit of their school, specialist teachers were asked to provide a brief description of the main difficulties or diagnosis (if known) and then to rate the pupil's difficulties in terms of extent of difficulty across five key areas of functioning: mobility, hearing, vision, language, and learning. For each area of functioning, specialist teachers were asked to rate severity using a 4-point scale: 0 = no impairment, 1 = mild impairment, 2 = moderate impairment, and 3 = severe impairment. Summing these ratings for each pupil generated a total disability score, which ranged from a possible minimum of 1 to a possible maximum of 15. These are reported in Appendix 1.

### Educator ratings of pupil interaction with technology

Four educations were asked to rate the 2-min video recording of each pupil with SEND interacting with the technology. Two raters were from Malawi and two were from the UK. For each country, one rater was a teacher of early years pupils and the other was an academic specializing in SEND (first and second author). This afforded comparison of ratings across countries and professions. Each rater was given access to the series of video recordings obtained for the sample of pupils with SEND in this study. To control against order effects influencing ratings each of the four raters was given a specific order in which to view the video recordings and the order was randomized across raters. They were also sent a form to record their ratings for each pupil (see Appendix 2). Raters were asked to categorize, independently, a set of behaviors reflecting different interactions with the technology and maths apps that they observed in each of the 2-min videos. Response categories referred to the frequency with which a particular behavior was observed as specified by the following 5-point likert scale: 1 = Rarely (0–20%), 2 = Not Often (21–40%), 3 = Sometimes (41–60%), 4 = Often (61–80%), and 5 = A Lot (81–100%). For each pupil, raters were asked a give categorical response using this scale to each of six different behaviors required for successful interaction with the apps, namely attention to task, motor precision (ability to touch a target object displayed on the screen), motor coordination (ability to drag and drop an object to a specific location on the screen), speed of response, response accuracy, and enjoyment with the app. Raters were given 3 weeks to complete this task.

### Monitoring data

A standard feature of the maths apps employed in this study is that time spent on task and number of topics passed (certificates awarded) for each session is recorded for each pupil when they use the technology. These data are sent via servers in each participating school to the app developers, *onebillion*, in the UK, who made these data available to us for analysis. Accordingly, we were able to utilize these monitoring data to generate a measure of rate of progress (time spent on task/topics passed) for each pupil with SEND and compare this to the average rate of progress for mainstream pupils in standard 1 from the larger Unlocking Talent Project (see above) who were receiving this technology intervention as part of their usual maths instruction. As the mainstream pupils were in standard 1, they were being taught the same mathematics curriculum as the sample SEND pupils, who were all following the standard 1 mathematics curriculum.

To further validate topics passed (certificates awarded) within the apps and progress rate (time spent on task/topics passed) as a measure of learning we correlated topics passed and progress rate with performance gains (difference between pre and post intervention) on a standardized measure of mathematical ability, the Early Grade Mathematics Assessment (EGMA) (USAID, 2011), administered individually to a group of 116 mainstream standard 1 pupils participating in the larger Unlocking Talent Project that is currently taking place in Malawi (see previous section). Pupils were given EGMA before and after intervention with the *onebillion* maths apps. On average, pupils received the intervention for 16 h. Results showed that topics passed within the apps correlated significantly with EGMA performance gains, *rs* = 0.24, *p* = 0.009 demonstrating that over the duration of the intervention, the more topics a pupil passed (certificates awarded) the more their maths ability improved. A similar correlation was found for progress rate (minutes taken to complete a topic). A significant negative correlation was found for this group of mainstream pupils between progress rate and EMGA gains, *rs* = −0.22, *p* = 0.021, demonstrating that the faster pupils progressed through the topics the more their maths ability improved. Thus, these results show criterion-related concurrent validity for using topics passed (certificates awarded) and rate of progress as a measure of learning in this study.

### Data analyses

Where data permitted, all analyses were performed with the whole sample of SEND pupils at a two-tailed level of probability with *p* = 0.05. A series of analyses were conducted to address the three key questions posed.

### How do pupils with send interact with the tablet technology and maths apps?

To address this question, educator ratings of the pupils with SEND interacting with the technology were correlated with the total disability scores provided by the specialist teachers. First, to establish reliability of the educator ratings generated from the 2-min video recordings, inter-rater reliability was compared across pairs of raters within Malawi and the UK (early years teacher and academic) and then within professions across countries (early years teachers from Malawi and the UK, and academics from Malawi and the UK), using Cronbach's Alpha (Cronbach, 1951). Second, to establish the extent to which pupils with SEND could interact with the technology, mean ratings were calculated across the four educators for each of the six behaviors measured through the video recordings. Then, to investigate if ratings from the 2-min video recordings differed across countries and between the six behavioral interactions measured, a 2 (country) × 6 (behavior) Chi-Square Test of Independence was conducted with summed frequency ratings from each pair of raters, per country per behavior. Third, to explore how extent of disability related to the ability of SEND pupils to interact with the tablet technology and maths apps a Pearson's Product Moment Correlation was conducted using the total disability score provided by the specialist teachers and the summed ratings given by the four educators to each of the behavioral interactions measured through the videos.

### Can pupils with send make progress in learning maths with the apps?

Monitoring data provided by *onebillion* was used to assess if pupils with SEND can learn with this technology. The total number of topics completed by each SEND pupil was considered first. Then, to determine progress rate (minutes per topic) for each pupil with SEND, total time on task (minutes) was divided by total number of topics passed. This accounted for any differences in the number of sessions that each pupil had attended within the pupil sample. Progress rate was also calculated for each of the mainstream pupils from standard 1 and standard 2 at LD and SJD who were receiving maths instruction with the tablet technology as part of their usual practice. Mean progress rate was then calculated and compared across the sample of SEND and mainstream pupils.

### How does extent of disability and interactions of pupils with send with the technology relate to their progress in learning maths?

To address this question, progress rate for learning maths within the apps was correlated with (i) total disability scores provided by specialist teachers and (ii) summed ratings across the four educators for each of the six behaviors assessed through the 2-min video recordings of pupils with SEND interacting with the technology and apps, through a series of Pearson's Product Moment Correlations. In addition, a stepwise regression was conducted with progress rate as the dependent variable and any significant behavioral measure as a predictor variable entered at each step in order of strength of correlation.

## Results

Table 1 shows that 1.04% of pupils were registered with SEND. There were significantly more boys (*N* = 21) than girls (*N* = 12) registered with SEND (binomial test, *p* = 0.041), and the gender distribution across schools did not differ significantly (χ^2^ = 0.73, *p* = 0.392). There was no significant difference between schools for pupils with SEND in terms of age of pupils [*t*_(31)_ = 1.42, *p* = 0.165], years in school [*t*_(24.13)_ = 1.26, *p* = 0.220], or total disability score [*t*_(26.65)_ = 0.98, *p* = 0.335], so all of the following analyses were conducted with the whole SEND pupil sample (*N* = 33).

### How do pupils with send interact with the tablet technology and maths apps?

It was not possible to make video recordings of two pupils (IK/LD/S1 and KB/LD/S1) within the timeframe available on the day. In addition, two recordings (AG/LD/S1 and JV/LD/S1) were not suitable for analysis because they failed to capture the pupil's interactions with the technology. Furthermore, one Malawi rater did not provide a full set of ratings for two pupils (GM/LD/S1 and EM/LD/S1) and one UK rater did not provide a full set of ratings for another pupil (MM/LD/S1). This resulted in a total sample of 26 video recordings will a full set of ratings across all four raters. Chronbach's alpha revealed a high degree of consistency between pairs of raters from Malawi [α_(27)_ = 0.64, *p* = 0.006] and the UK [α_(28)_ = 0.79, *p* < 0.001], and across the two early years teachers [α_(28)_ = 0.87, *p* < 0.001] and the two academics [α_(27)_ = 0.60, *p* = 0.011]. This demonstrates that the ratings generated from the 2-min video recordings of pupils with SEND interacting with the tablet technology and maths apps were reliable.

Table 2 reports mean ratings across the four educators for each of the six behaviors measured through the 2-min video recordings of SEND pupils interacting with the tablet technology and maths apps. As can be seen, most behaviors received a mean rating of between 3 (Sometimes: 41–60%) to 4 (Often: 61–80%), and the average rating across all behaviors was 3.40, indicating that SEND pupils can interact with this technology, unassisted, for the majority of time.

**Table 2:** Mean rating for each of the six behavioral interactions with the maths apps by pupils with SEND (*N* = 26).

**Descriptive statistics**	**Behavior**					
	**Attention to task**	**Motor precision**	**Motor coordination**	**Speed of processing**	**Response accuracy**	**Enjoyment with app**
Mean (SD)	3.88 (0.93)	3.66 (0.96)	3.64 (0.91)	3.45 (0.93)	2.80 (1.32)	2.96 (0.99)
Min–Max	1.00–5.00	1.00–5.00	1.25–5.00	1.00–5.00	1.00–5.00	1.00–5.00

Furthermore, results of a 2 (country) × 6 (behavior) Chi Square Test of Independence were not significant (χ^2^ = 3.58, *p* = 0.612), illustrating the degree of interaction with the tablet technology by the SEND pupils was similar for the six behavioral measures rated by Malawi and the UK educators. Finally, the correlation between total disability score and summed ratings for interactions of SEND pupils with the apps was not significant [*r*_(26)_ = −0.36, *p* = 0.074], although the direction of correlation indicated that pupils with lower disability scores interacted with the apps to a greater extent than more severely impaired pupils.

### Can pupils with send make progress in learning maths with the apps?

Monitoring data provided by *onebillion* was not available for one pupil (MM/LD/S1) so analyses were conducted with 32 pupils. Inspection of the monitoring data revealed that all of the pupils with SEND completed at least one topic covered in the maths apps and the mean number of topics completed by the group was 4.44 (*sd* = 3.98, min–max = 1–18). This demonstrates that on average the group of SEND pupils had completed 11.68% of the maths course. In addition, the mean progress rate (minutes per topic) for the pupils with SEND was 234 mins (*sd* = 180, min–max 38–704), illustrating that the average time taken to complete a topic was around 4 h. In contrast, the mean progress rate of a large group of mainstream pupils from standard 1 taking part in the Unlocking Talent Project (*N* = 116) was 127 mins (*sd* = 73, min–max = 38–444), demonstrating that mainstream pupils following the same curriculum take on average around 2 h to complete a topic. Thus, these results reveal that pupils with SEND can learn basic mathematics with this technology but they progress at half the rate of mainstream pupils.

### How does extent of disability and interactions of pupils with send with the technology relate to their progress in learning maths?

Pearson's Product Moment Correlations revealed a significant relationship between progress rate of SEND pupils in learning maths through the apps and their total disability score [*r*_(32)_ = 0.38, *p* = 0.033]. This positive correlation illustrates that pupils with a low disability score take less time to complete a topic in the maths apps than more impaired pupils. In addition, a significant negative relationship was found between attention to task by SEND whilst interacting with the apps and their progress rate [*r*_(26)_ = −0.40, *p* = 0.045], illustrating that pupils who progressed faster through the apps attended to task more often than those who made slower progress. None of the other correlations with behavioral ratings and progress rate were significant although all were negative indicating that pupils with fewer disabilities interacted more with the apps than pupils with more severe difficulties [total interactions: *r*_(26)_ = −0.31, *p* = 0.130, motor precision: *r*_(26)_ = −0.15, *p* = 0.479, motor coordination: *r*_(26)_ = −0.11, *p* = 0.594, speed of processing: *r*_(26)_ = −0.27, *p* = 0.176, response accuracy: *r*_(26)_ = −0.36, *p* = 0.072, enjoyment with app: *r*_(26)_ = −0.29, *p* = 0.147].

To investigate the unique contribution that attention to task ratings and total disability score made to the prediction of progress rate with the apps by SEND pupils a stepwise regression was performed. Table 3 summarizes the results from this stepwise regression, where progress rate was the dependent variable and attention to task ratings and total disability score were entered as predictor variables at step 1 and step 2, respectively. As can be seen, both models were significant (model 1: *F* = 5.45, *p* = 0.028, model 2: *F* = 6.69, *p* = 0.005) and explained 18.5 to 36.8% of the variance. Significant improvements to the model were found at step 2 when adding in total disability score (Δ*R*^2^ = 0.18, *p* = 0.017). While attention to task ratings were a significant predictor in model 1 (*p* = 0.028), their contribution was no longer significant when total disability score was added at step 2 (*p* = 0.111). Thus, for pupils with SEND, specialist teacher ratings of extent of disability were the best predictor of their ability to progress in learning basic mathematics with the tablet technology and interactive apps evaluated in this study.

**Table 3:** Summary of stepwise regression for attention to task ratings of pupils with SEND and total disability score in predicting their progress rate in mathematics.

**Summary of results**	**Step, variable(s)**	
	**1**	**2**
	**Attention to task ratings**	**Attention to task ratings** + **Total disability score**
Model	*R* = 0.43, *R*^2^ = 0.19	*R* = 0.61, *R*^2^ = 0.368
Significance	*F* = 5.45, *df* (1, 24), *p* = 0.028	*F* = 6.65, *df* (1, 23), *p* = 0.005
Change	Δ*R*^2^ = 0.19, Sig. Δ*F* = 0.028	Δ*R*^2^ = 0.18, Sig. Δ*F* = 0.017
Unstandardized coefficients	*B* = −21.52, Std. Error = 9.22	*B* = −14.46, Std. Error = 8.73 *B* = 32.39, Std. Error = 12.56
Standardized coefficients	Beta = −0.430	Beta = −0.289 Beta = 0.450
Significance	*t* = −2.335, *p* = 0.028	*t* = −1.656, *p* = 0.111 *t* = 2.578, *p* = 0.17

As the composite measure of extent of disability was comprised of five areas of functioning (mobility, hearing, vision, language, and learning), further exploratory analyses were conducted to investigate if specific areas of functioning correlated with progress rate. Data were not normally distributed, so a series of Spearman's Rank Correlations was conducted. Results showed significant positive correlations between progress rate and teacher ratings for hearing, *r*s_(32)_ = 0.47, *p* = 0.007, and language, *r*s_(32)_ = 0.38, *p* = 0.033, demonstrating that pupils with more severe difficulties with hearing and/or language took longer to progress through the apps than pupils with relatively good hearing and language skills. None of the other specific areas of functioning correlated significantly with progress rate: mobility, *r*s_(32)_ = −0.14, *p* = 0.433, vision, *r*s_(32)_ = 0.05, *p* = 0.792, learning, *r*s_(32)_ = 0.020, *p* = 0.914.

## Discussion

Reported here is a novel investigation into the learning gains attained by pupils with SEND when using touch-screen tablets and interactive apps designed to support the acquisition of basic mathematical skills. Whilst previous research has reported that SEND pupils respond well to tablet technology and engage highly with the learning task (Strnadova and Cumming, 2013; Chmiliar, 2017; Khoo, 2017), few studies have assessed if this technology is effective at raising learning attainment for pupils with SEND using quantitative techniques. Most educational apps host a suite of features that can enhance learning for mainstream pupils (see also Rose et al., 2005; Condie and Munro, 2007), but it has yet to be established if these multisensory, interactive, features will support skill acquisition for pupils with a range of physical, sensory, and learning difficulties. Although assistive aids, either internal or external to the technology, can facilitate the use of tablet technology and interactive apps for SEND pupils (Dell et al., 2017), in the current study SEND pupils accessed the technology without the use of additional assistive technology aids. Specifically, the reliance on verbal instructions and visual displays within the apps evaluated in this study, as well as the need for precise hand-eye coordination to identify, move, and trace objects on the touch-screen, might pose particular problems for SEND pupils with sensory processing and/or motor coordination difficulties.

We observed a group of 33 pupils registered at a SEND unit attached to one of two mainstream primary schools that are implementing a novel touch-screen tablet technology intervention as part of the Unlocking Talent Project that is currently being roled out across Malawi. Results showed that each of the SEND pupils could interact to some extent with the tablet technology and maths apps, when delivered without additional assistive technology aids. Furthermore, each SEND pupil had made some progress in learning basic mathematics with this technology intervention, as all pupils with SEND had passed at least one topic included in the apps. By using monitoring data collected within the apps from each session we were able to quantify progress rate (time taken to learn a topic) for each pupil. Our data showed that on average SEND pupils took around 4 h to complete a topic within the maths apps, which was twice as long as mainstream pupils in the first year of schooling, which is the same ability level (standard) as most of the SEND pupils. These results highlight that this tablet technology intervention is effective for supporting the acquisition of basic mathematical skills for pupils with SEND albeit at a slower rate than mainstream pupils. Our results demonstrate clearly that SEND pupils can learn basic mathematics with this tablet technology. Within our sample of SEND pupils, 11/33 pupils had been in school for more than 1 year illustrating that they had not made progress within the conventional classroom learning environment and had thus had to repeat grades. Our results demonstrate that given appropriate tools SEND pupils can learn basic mathematics. This is an important message for government, educators, and parents, in a country where a commonly held belief is that these children have limited learning capability. Our results indicate that tablet technology can provide an alternative means of education for these pupils that could raise learning outcomes and encourage parents to send their children with SEND to school, rather than keeping them at home (Mutua and Dimitrov, 2001), and could help to change attitudes toward individuals with SEND in terms of their ability to learn.

Whilst our results illustrate that SEND pupils can learn basic mathematical skills with this technology intervention, it was not possible to compare their progress rate with the technology intervention to that of standard teacher-led mathematics instruction. Firstly, reliable monitoring data of time on task and skill acquisition is not available from standard teacher-led practice so attainment is usually assessed through administering standardized tests, such as the Early Grade Mathematics Assessment (USAID, 2011), and many of the pupils with SEND in our sample would have encountered difficulty in completing this type of standardized assessment, rendering it unsuitable for these pupils. However, the Malawi education system allows pupils that fail to reach the expected level of attainment within a school year to repeat that grade the following year, so repetition rates can provide an indication of ability to progress with standard teacher-led instruction. Within our sample of SEND pupils, 11/33 pupils had been in school for more than 1 year yet all were following basic mathematics instruction provided in the first year of schooling. This suggests that at least a third of the pupils with SEND in our study had failed to reach the expected level of attainment in mathematics taught over the first year of school with usual teacher-led instruction so were repeating this grade. Furthermore, a pupil-level randomized control trial with mainstream pupils in Malawi compared directly mathematics instruction with the technology intervention (intervention group) to standard teacher-led practice (control group) over a specified period of time and revealed significantly greater learning gains for pupils who received the tablet technology intervention than those who received standard teacher-led practice (Pitchford, 2015). As most pupils with SEND could interact with the tablet technology across the six behaviors measured in this study, this raises the possibility that rate of progress for SEND pupils might also be faster through instruction with the technology intervention compared to standard teacher-led practice, although this needs to be confirmed in a full systematic investigation before any firm conclusions can be drawn.

Results from the correlational analysis revealed that extent of disability and attention to task significantly predicted the rate of progress by which pupils with SEND acquired basic mathematical skills with the tablet technology intervention, accounting for just over a third (37%) of the variance. In particular, extent of disability was the strongest predictor of progress rate. This indicates that this technology-based intervention is most suited to pupils with mild-moderate difficulties and suggests that children with severe difficulties might struggle to learn basic mathematics with this technology alone, without the use of additional assistive technology aids. Pupils with a high level of disability may require alternative instruction targeted to accommodate their specialized needs. The finding that rate of progress is associated to extent of disability is however predictable and validates the new rating scale for measuring extent of disability that was developed in this study (Appendix 1). This rating scale could be used by specialist teachers considering to implement this technology intervention within their SEND classes as guidance for which pupils with SEND might benefit most.

The composite measure of extent of disability captured multiple difficulties that pupils with SEND often experience. Whilst there was limited variance within each of the five areas of functioning measured by the teacher ratings comprising the total disability score, exploratory analyses were conducted to investigate if particular areas of functioning correlated with progress rate. Results showed that pupils with more severe difficulties with hearing and/or language took significantly longer to progress through the apps than pupils with relatively good levels of functioning in these two domains. Appendix 1 shows that pupils with moderate to severe hearing difficulties also tended to have some degree of language difficulties, as may be expected (e.g., Tierney et al., 2008). These results highlight a potential limitation of the utility of this intervention for SEND pupils with moderate to severe hearing and/or language skills without further adaptation, such as providing instructions through sign language within the apps instead of giving verbal instructions. These results should be treated with caution due to the limited variation of ratings within each area of functioning. A larger study is required adopting the unique methodology employed in this study to determine which areas of functioning are most predictive of learning with this technology.

However, qualitative inspection of details given by specialist teachers of SEND pupils in Appendix 1 corroborates these exploratory correlations. Children rated by their teachers as having the most difficulties tended to have communication problems because they were either deaf and/or mute or motor difficulties related to cerebral palsy. Our video recordings showed that pupils with cerebral palsy could interact with the touch-screen tablets and maths apps if they had some degree of fine manual control which could enable them to isolate specific objects on screen and drag and drop objects to different screen locations as required to navigate through the apps. Increasing the “catchment” area for object movements within the apps might assist these pupils to engage with the apps more easily. Previous qualitative studies of pupils with cerebral palsy using touch-screen tablet technology have highlighted how particular features of interactive apps can support both physical functioning and learning needs (Khoo, 2017). In contrast, pupils with severe auditory difficulties struggled to interact with the apps because they could not hear the verbal instructions given by the virtual teacher which was necessary to complete the topics and quizzes. Clearly, adaptations to the software are required for pupils with severe sensory impairments to access this technology fully, such as inclusion of sign language prompts instead of spoken language instructions within the apps. Similarly, pupils with severe visual impairments struggled to learn with these apps, as the content presentation relies to a large extent on visual processing. Although the correlation between progress rate and vision was not significant, this probably reflects the limited variation in teacher ratings for visual impairments across the current sample of SEND pupils. A larger study is needed to establish more conclusively if these apps are problematic for pupils with severe visual impairments. External assistive technology aids could be used in conjunction with the apps to facilitate engagement and learning with this tablet technology intervention (Dell et al., 2017) although this will require additional investment and might restrict using the technology alongside mainstream peers in an inclusive educational setting. Despite these challenges, pupils with SEND seemingly enjoyed using the interactive maths apps evaluated in this study, as evidenced by the mean video recording rating of 2.96 (which equates to between 41 and 60% of the time). This corroborates previous qualitative reports that SEND pupils have positive experiences and high levels of engagement in learning with educational apps delivered on touch-screen tablets (Strnadova and Cumming, 2013; Chmiliar, 2017; Khoo, 2017) and could encourage these pupils to continue attending school if used on a regular basis.

As with class teachers of mainstream pupils, the specialist teachers found this intervention easy to implement with SEND pupils as the apps provide one-to-one tutoring that is not possible within conventional teaching methods in Malawi. The apps also encouraged greater parental involvement with SEND pupils education than is typical for usual classroom lessons, as several of the pupils' parents volunteered to assist in the Learning Center during the sessions with the SEND pupils, especially at LD school. Support from parents, VSO volunteers, and specialist teachers focussed on resolving technical issues with the tablets, such as checking the volume control, but also some pedagogical support was offered offline, by way of explaining concepts being taught within the apps. Importantly, emotional support was given to the SEND pupils, as the adults encouraged them to touch the arrow to progress to the next trial. In addition, praise and celebrations were given by the adults when a SEND pupil passed a quiz, enhancing the enjoyment of learning with this technology. Increased parental involvement in SEND pupils' education could be an additional benefit to this technology intervention as it fosters greater school-home links, and secondary benefit could even be to raise numeracy skills in the parents of SEND pupils through their assistance in implementing this intervention, given the high levels of innumeracy within the Malawi population (Kadzamira and Rose, 2003).

If the tablets could be made available to pupils with SEND to use at home, either after school or at weekends, this might foster even closer school-home links (Strnadova and Cumming, 2013) which, in turn, may engender positive attitudes from parents and other community members toward pupils with SEND and their capability to learn. A potential risk to this possibility concerns security, as devices have been stolen when introduced to support school-home links in some countries and this is a concern to low-income families as they do not have the ability to pay for any losses incurred (Katz and Gonzalez, 2016). The use of iPads in this intervention could pose a particular security risk as these tablets are expensive and desirable objects. The app developers, *onebillion*, are exploring alternative devices that are more affordable than iPads but do not compromise the effectiveness of delivering this intervention. Clearly, hardware cost is a key factor to consider when scaling an intervention such as this. In addition, if the SEND pupils use the tablets and apps at home without the support of an engaged adult the effectiveness may be reduced if technical and emotional support is removed. Given the parents interest in assisting with implementing this technology intervention in schools, it is more likely that parents would be highly motivated to support their SEND pupils working with these apps in the home environment, should they be given the opportunity. As the tablets can be charged at school reliably by solar panels, this could provide a dependable school-home link for SEND pupils, and foster greater community involvement in their education.

In the current study, SEND pupils accessed the technology intervention in separate sessions from mainstream pupils at each participating school, but to promote greater inclusive education, sessions within the Learning Centers could include both SEND and mainstream pupils. Greater integration of SEND and mainstream pupils across the primary school years through the use of tablet technology might also serve to breakdown some of the sociocultural barriers that prevent pupils with SEND from participating fully in primary school education and reaching their full learning potential. For example, it might encourage parents to send or to keep their children with learning difficulties in school. Interestingly, our data revealed that the overall prevalence of pupils with SEND within our sample was 1.04% which is less than the national average of 2.3% (Munthali, 2011). However, this was driven mainly by a very low prevalence of SEND pupils at the urban primary school (LD = 0.67%) compared to the rural primary school (SJD = 2.16%) where the prevalence was similar to the national level. This suggests that some children with SEND in urban areas are being kept at home. Tablet technology could address this apparent inequality in access to high-equality educational instruction by embedding the technology intervention within community settings and providing a bridge between the home and school environment. Furthermore, the intervention does not require specialist teaching to implement successfully, so this might address the shortage of specialist teachers within the Malawi education system to some extent (Hughes et al., 2016), as non-specialist teachers should be able to implement this intervention with SEND pupils.

To conclude, this quantitative study provides “proof of concept” that interactive apps delivered on touch-screen tablets can be an effective means of accessing high-quality educational instruction for pupils with SEND and raising learning outcomes. Whilst most pupils with mild to moderate learning difficulties can interact with this technology its utility might be limited for more severely impaired pupils, especially those with sensory processing and/or fine motor difficulties. We recommend that primary schools consider supplementing specialist teacher-led instruction with tablet technology interventions shown to be effective at raising learning outcomes for pupils with SEND to “ensure inclusive learning and equitable quality education and promote lifelong learning for all” (United Nations, 2016, p. 5).

## Author contributions

NP and PH designed and conducted the study and analyzed the data. EK and AC provided background on the context of SEND education in Malawi and AC processed ethical approval for the study as part of the Unlocking Talent Project. NP wrote the article. All authors reviewed and commented on the final article text.

### Conflict of interest statement

The authors declare that the research was conducted in the absence of any commercial or financial relationships that could be construed as a potential conflict of interest.
