# Supplementary material for: Interactive Apps Promote Learning of Basic Mathematics in Children With Special Educational Needs and Disabilities
Source: Front Psychol. 2018 Mar 6;9:262. doi: 10.3389/fpsyg.2018.00262 (PMC5845689; doi:10.3389/fpsyg.2018.00262)
Supplement: Supplementary file 1 [file DataSheet1.docx]

**Appendix 1. Participant details for the total sample (N = 33) drawn from two SEN units (LD N = 16, SJD N = 17). SEN teacher ratings for each child on M = mobility, H = hearing, V = Vision, Lang = language, Learn = learning in terms of degree of impairment: Severe = 3, Moderate = 2, Mild = 1, None = 0; Total Disability Score = 1-15. *Children without video-recordings using tablet technology**

| **Child/School/**  **Standard** | **Gender**  **(M/F)** | **Age in years** | **Years in**  **School** | **Diagnosis/**  **Difficulties** | **M** | **H** | **V** | **Lang** | **Learn** | | **Total**  **Disability Score** |
| --- | --- | --- | --- | --- | --- | --- | --- | --- | --- | --- | --- |
| PM/SJD/S1 | M | 8 | 1 | Emotional and behavioural difficulties: aggressive, poor memory | 0 | 0 | 0 | 0 | | 1 | 1 |
| YM/SJD/S1 | F | 10 | 2 | Learning difficulty: difficulty in processing information, poor memory | 0 | 0 | 0 | 0 | | 1 | 1 |
| AK/SJD/S1 | M | 6 | 1 | Vision loss: sees only at an angle in periphery | 0 | 0 | 2 | 0 | | 0 | 2 |
| GM/SJD/S1 | M | 8 | 1 | Communication difficulties: low hear, soft voice, social difficulties | 0 | 1 | 0 | 0 | | 1 | 2 |
| GG/SJD/S1 | M | 8 | 2 | Learning difficulty: stutters, cannot concentrate on tasks, attention deficit, rushes to complete task | 0 | 0 | 0 | 1 | | 1 | 2 |
| FK/LD/S1 | F | 8 | 0.25 | Learning difficulties: very slow learner | 0 | 0 | 0 | 0 | | 2 | 2 |
| JL/SJD/S1 | F | 9 | 1 | Dyslexia: stutters, cannot process information, poor memory | 0 | 0 | 0 | 1 | | 1 | 2 |
| JF/SJD/S1 | F | 9 | 1 | Learning difficulties: stutters, cannot follow instructions | 0 | 0 | 0 | 1 | | 1 | 2 |
| LS/SJD/S1 | M | 9 | 1 | Communication difficulties: stutters, difficulties with articulation, poor memory | 0 | 0 | 0 | 1 | | 1 | 2 |
| CP/SJD/S1 | F | 10 | 2 | Learning difficulty: performs below average, poor concentration | 0 | 0 | 0 | 0 | | 2 | 2 |
| IK/LD/S1* | F | 10 | 4 | Learning difficulties: socially good but very slow learner | 0 | 0 | 0 | 0 | | 2 | 2 |
| GL/SJD/S1 | M | 11 | 3 | Learning difficulties: lacks selective attention, difficulty in finishing tasks in time | 0 | 0 | 0 | 0 | | 2 | 2 |
| KB/LD/S1* | M | 8 | 3 | Learning difficulties: epilepsy, petit mal seizure once a month, reduced verbal expression and comprehension | 0 | 0 | 0 | 1 | | 2 | 3 |
| JV/LD/S1 | F | 12 | 6 | Learning difficulties: can comprehend and express oral language a little but poor writing, slow learner | 0 | 0 | 0 | 2 | | 1 | 3 |
| HE/LD/S1 | M | 8 | 4 | Learning difficulties: little verbal comprehension and expression, very slow learner | 0 | 0 | 0 | 2 | | 2 | 4 |
| EP/LD/S1 | M | 12 | 0.25 | Communication difficulties: poor comprehension and poor expression, drools, can use pen but writing does not to make sense, low mood (apathy) | 0 | 0 | 0 | 2 | | 2 | 4 |
| MK/SJD/S1 | M | 16 | 3 | Cerebral palsy: difficulties with mobility and articulation | 2 | 0 | 0 | 1 | | 1 | 4 |
| MM/LD/S1 | M | 6 | 2 | Learning difficulties: can comprehend and express verbal language just a little, very slow learner | 0 | 0 | 0 | 3 | | 2 | 5 |
| CM/LD/S2 | M | 8 | 1 | Cerebral palsy: cannot walk, uses a wheelchair and walking frame, but can use both arms | 3 | 0 | 0 | 2 | | 0 | 5 |
| CF/LD/S1 | M | 13 | 3 | Learning difficulties: can comprehend verbal language a little but speaks in single words only, no sentences, very slow learner | 0 | 0 | 0 | 3 | | 2 | 5 |
| GM/LD/S1 | F | 13 | 0.25 | Learning difficulties and hyperactivity: verbal comprehension language is good but difficulties with expression, slow learner | 0 | 0 | 0 | 2 | | 3 | 5 |
| EM/LD/S1 | F | 18 | 4 | Downs Syndrome: has some verbal comprehension and expression but written language is very poor, very slow learner | 0 | 0 | 0 | 2 | | 3 | 5 |
| KM/LD/S1 | M | 10 | 5 | Communication difficulties: little comprehension and expression, cannot write, very slow learner | 0 | 0 | 0 | 3 | | 3 | 6 |
| OM/LD/S1 | M | 14 | 7 | Cerebral palsy: walks with difficulty, moderate use of arms, very strong, no speech but vocalises, good drawing skills, language preventing progress | 2 | 0 | 0 | 3 | | 1 | 6 |
| AG/LD/S1 | M | 8 | 1 | Cerebral palsy: cannot walk but can use both arms | 3 | 0 | 0 | 2 | | 2 | 7 |
| JM/SJD/S1 | M | 8 | 3 | Communication difficulties: mobility and articulation difficulties | 2 | 2 | 0 | 2 | | 1 | 7 |
| HJ/SJD/S1 | M | 9 | 2 | Communication difficulties: cannot hear or speak, difficulty staying in seat, difficulty following instructions | 0 | 3 | 0 | 3 | | 1 | 7 |
| SC/SJD/S1 | F | 13 | 6 | Autism: social isolation, difficulty following instructions | 0 | 1 | 0 | 3 | | 3 | 7 |
| WM/LD/S6 | F | 13 | 3 | Cerebral palsy: uses a wheelchair, can comprehend language but has poor verbal expression, slow learner | 3 | 0 | 0 | 2 | | 2 | 7 |
| TE/SJD/S1 | M | 7 | 2 | Communication difficulties: cannot hear or speak, cannot concentrate on class work, hyperactive | 0 | 3 | 0 | 3 | | 2 | 8 |
| JP/SJD/S1 | M | 8 | 1 | Cerebral palsy: difficulties with articulation, aggressive | 1 | 2 | 0 | 3 | | 2 | 8 |
| TM/LD/S1 | F | 8 | 0  (Just started) | Cerebral palsy: cannot walk, no wheelchair so being carried to school by mother, very high social deprivation, limited use of arms | 3 | 0 | 0 | 2 | | 3 | 8 |
| RK/SJD/S1 | M | 7 | 1 | Communication difficulties: cannot hear or speak, hyperactive, curved legs through Rickets | 1 | 3 | 0 | 3 | | 2 | 9 |
