# Supplementary material for: Interactive Apps Promote Learning of Basic Mathematics in Children With Special Educational Needs and Disabilities
Source: Front Psychol. 2018 Mar 6;9:262. doi: 10.3389/fpsyg.2018.00262 (PMC5845689; doi:10.3389/fpsyg.2018.00262)
Supplement: Supplementary file 2 [file DataSheet2.docx]

**Appendix 2. Example form used to record ratings of SEN pupils’ interactions with maths app as captured by 2-minute video recordings**

**E.G. Malawi Rater 1**

Thank you for agreeing to rate these videos of Special Educational Needs children in Malawi using the *onebillion* maths apps in a learning centre at their local primary school.

To complete your ratings please open each video, in the order provided. For each 2-minute video, first watch the video and then make your ratings using the response categories below. Enter just one response category for each of the behaviours listed in the form. After you have made your ratings, close the video, and then open the next video from your list.

Repeat until you have rated all of the videos in your file.

| **Response Category** | | | | |
| --- | --- | --- | --- | --- |
| **1**  **Rarely**  **0-20%** | **2**  **Not Often**  **21-40%** | **3**  **Sometimes**  **41-60%** | **4**  **Often**  **61-80%** | **5**  **A Lot**  **81-100%** |

| **Child**  **ID** | **Behaviour to Rate** | | | | | |
| --- | --- | --- | --- | --- | --- | --- |
|  | **Attention to Task** | **Motor Precision** | **Motor Coordination** | **Speed of Processing** | **Response**  **Accuracy** | **Enjoyment with App** |
| **Example** | **5** | **2** | **2** | **1** | **3** | **4** |
|  |  |  |  |  |  |  |
|  |  |  |  |  |  |  |
|  |  |  |  |  |  |  |
|  |  |  |  |  |  |  |
|  |  |  |  |  |  |  |
|  |  |  |  |  |  |  |
|  |  |  |  |  |  |  |
|  |  |  |  |  |  |  |
|  |  |  |  |  |  |  |
|  |  |  |  |  |  |  |
|  |  |  |  |  |  |  |
|  |  |  |  |  |  |  |
|  |  |  |  |  |  |  |
|  |  |  |  |  |  |  |
|  |  |  |  |  |  |  |
